# Prediction of metal ion ligand binding residues by adding disorder value and propensity factors based on deep learning algorithm

**DOI:** 10.3389/fgene.2022.969412

**Published:** 2022-08-11

**Authors:** Sixi Hao, Xiuzhen Hu, Zhenxing Feng, Kai Sun, Xiaoxiao You, Ziyang Wang, Caiyun Yang

**Affiliations:** ^1^ College of Sciences, Inner Mongolia University of Technology, Hohhot, China; ^2^ Inner Mongolia Key Laboratory of Statistical Analysis Theory for Life Data and Neural Network Modeling, Hohhot, China

**Keywords:** metal ion ligand, deep neural network algorithm, disorder value, propensity factors, binding residues

## Abstract

Proteins need to interact with different ligands to perform their functions. Among the ligands, the metal ion is a major ligand. At present, the prediction of protein metal ion ligand binding residues is a challenge. In this study, we selected Zn^2+^, Cu^2+^, Fe^2+^, Fe^3+^, Co^2+^, Mn^2+^, Ca^2+^ and Mg^2+^ metal ion ligands from the BioLip database as the research objects. Based on the amino acids, the physicochemical properties and predicted structural information, we introduced the disorder value as the feature parameter. In addition, based on the component information, position weight matrix and information entropy, we introduced the propensity factor as prediction parameters. Then, we used the deep neural network algorithm for the prediction. Furtherly, we made an optimization for the hyper-parameters of the deep learning algorithm and obtained improved results than the previous IonSeq method.

## 1 Introduction

The interaction between proteins and ligands is particularly important for a variety of biological processes such as the transport of oxygen, the transfer of cellular signals, energy conversion and muscle contraction (Reif, 1992; Davis et al., 2004; Jeffrey et al., 2006). Therefore, it is valuable to accurately identify the protein-ligand binding site for understanding protein function, disease occurrence and molecular drug design (Laurie and Jackson, 2006). Among these ligands, more than one-third are metal ion ligands (Hu et al., 2020). Although the bond length, bond angle and torsion angle of each metal ion ligand binding to proteins are different, from the perspective of the spatial structure of protein binding to metal ion ligands, all metal ion ligands combine with residues on the “pocket” of the protein surface to form a complex and stable spatial structure. Therefore, we selected the eight metal ion ligands as a series of studies. Due to the small size and active chemical properties of metal ion ligands, it is a challenging work to predict the metal ion ligand binding residues with similar chemical structures by theoretical calculation methods.

In the prediction of protein-metal ion ligand binding sites, predecessors have done a lot of research work and made significant progress. At present, the feature parameters of most studies were based on the information of primary sequences, the physical and chemical and predicted structure information. For example, in 2016, Jiang et al. (2016) used the component of amino acids, the autocross covariance value, center motif and site conservative information as feature parameters for predicting the binding sites of Ca^2+^ ligands, and the total predict accuracy (Acc) was better than 70.0%. Then, Hu et al. (2016b) used position specific scoring matrix (PSSM), secondary structure, and the real values of phi and psi as feature parameters to predict the binding sites of Cu^2+^, Fe^2+^, Fe^3+^, and Zn^2+^, and the obtained Matthew’s correlation coefficient (MCC) was higher than 0.20, Acc was higher than 97.0%. In 2017, Cao et al. (2017) selected the sequences information, site conservative information, secondary structure information, matrix scoring values of the hydrophilic-hydrophobic and polarization charge as feature parameters to identify the binding sites of 10 metal ion ligands, the MCC value was higher than 0.502. In 2019, Wang et al. (2019) selected the component information and site conservative information of features such as amino acids, secondary structure, relative solvent accessibility, hydrophilic-hydrophobic, and polarization charge to predict binding sites of 10 metal ion ligands, the ACC was higher than 68.0%. Although the physicochemical feature of amino acid and predicted structure information were usually used as feature parameters in previous studies, the obtained prediction results by different extraction methods were also different. Therefore, the selection and extraction methods of feature parameters need to be emphasized and innovatively optimized in study.

In recent years, many traditional machine learning algorithms have been used to predict protein-metal ion ligand binding sites, such as support vector machine (SVM), random forest (RF), bayesian classifier. For example, in 2016, Hu et al. (2016a) developed a method called IonSeq based on SVM to predict 10 metal ion ligands, and the values of sensitivity (S_n_)_,_ Acc and MCC were higher than 5.57%, 74.09% and 0.1516, respectively. In 2020, Liu et al. (2020) used RF algorithm to predict the binding sites of 10 metal ion ligands, the MCC and Acc were better than 0.07 and 52%, respectively. In 2021, Wang et al. (2021) applied the SVM algorithm to predict ten metal ion ligands, the S_n_ and MCC values were greater than 39.5% and 0.118, respectively. Although these traditional algorithms obtained good results in the prediction of protein-metal ion ligand binding residues, it is difficult for them to learn deeply and effectively from the growing amount of data in the post-genomic and big data era (Song et al., 2020). At present, deep learning is a new way to realize machine learning, and has powerful deep learning capabilities and parallel distributed processing capabilities. It has been used in the study of protein-metal ion ligand binding residues, and good prediction results have been obtained (Cui et al., 2019).

In this paper, the deep neural network (DNN) algorithm was used to predict the binding residues of eight metal ion ligands (Lorenzo-Trueba et al., 2018). Based on protein sequence, we selected amino acids, secondary structure, relative solvent accessibility, dihedral angles, charge and hydrophilic-hydrophobic as basic feature parameters, and added disorder values as new feature parameters. On the basis of component information, position weight matrix, information entropy and propensity factors were added as a new feature parameter. By optimizing the three hyper-parameters in the deep learning algorithm, the prediction results have been significantly improved.

## 2 Materials and methods

### 2.1 Selection of data set

The data set is the basis of prediction. To ensure the authenticity of data and the accuracy of experiment, we selected eight metal ion ligands from the BioLip database: Zn^2+^, Cu^2+^, Fe^2+^, Fe^3+^, Co^2+^, Mn^2+^, Ca^2+^, Mg^2+^ (Yang et al., 2013). In order to construct non-redundant data set, we filtered the data samples by eliminating the sequence length of less than 50 amino acids, resolution greater than 3 Å, and the sequence identity higher than 30%. The fragments were intercepted on the protein sequence by using the sliding window method. To make that every residue of the protein chain appears in the center of the fragment, we added (L−1)/2 pseudo-amino acids at both ends of the protein chain. Here the length L of the intercepted fragments was taken according to references (Hu et al., 2016a). If (L+1)/2 is a binding residue, it is defined as a positive fragment, otherwise it was a negative fragment. The non-redundant data set of eight metal ion ligands is shown in Table 1.

**TABLE 1:** The non-redundant data set for eight metal ion ligands.

Ligands	L	Chains	P	N
Zn^2+^	13	1,428	6,408	405,113
Cu^2+^	15	117	485	33,948
Fe^2+^	9	92	382	29,345
Fe^3+^	11	217	1,057	68,829
Co^2+^	11	194	875	55,050
Mn^2+^	11	459	2,124	156,625
Ca^2+^	9	1,237	6,789	396,957
Mg^2+^	15	1,461	5,212	480,307

Note: Ligands represents metal ion ligand; L represents the sequence fragment length; Chains represents the number of chains in a protein; P represents the binding residues; N represents the non-binding residues.

### 2.2 Selection of feature parameters

#### 2.2.1 Introduction of new feature parameter

**FIGURE 1:**
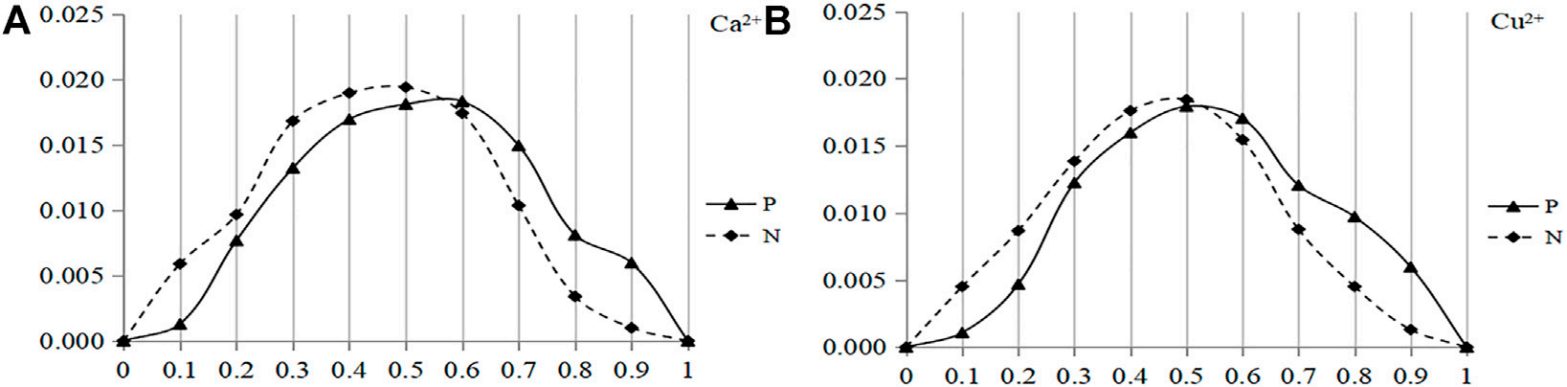
Distribution of disorder values of the binding residue and non-binding residue of Ca^2+^ and Cu^2+^ ligands. Note: The ordinate is the probability of the disorder value; P, represents the binding residues; N, represents the non-binding residues.

Note: The abscissa is the disorder value; the ordinate is the probability of the disorder value.; Solid line and dotted line are the positive and negative sets, respectively.

In recent years, researchers have discovered a special class of amino acid fragments in protein sequences. Due to the fact that these fragments lack stable structure and are highly variable, they are called the disordered regions of proteins (Dunker et al., 2002). The instability and high variability of these disordered regions can lead to their easy interaction with ligands (Noivirt-Brik et al., 2009). In this way, it has been applied to the prediction of protein-protein interaction, and good prediction results have been obtained (Zhang et al., 2016). In this work, we used the IUPred2A software and converted the structural state of each amino acid in the protein sequence into the disorder score (Mészáros et al., 2018; Gábor and Dosztányi, 2020). The disorder score ranges from 0 to 1, and the higher the value, the more disordered the structure of amino acids. In this paper, the disorder values of positive (negative) set fragments were statistically analyzed, since the disorder value was continuous, it was divided into 10 intervals for the convenience of statistics. Taken Ca^2+^ and Cu^2+^ as examples, the distribution of disorder value of the binding residue and non-binding residue was shown in Figure 1.

It can be seen from Figure 1A that the difference of the disorder values of Ca^2+^ ligand between the positive and negative sets was mainly concentrated in two intervals: 0–0.55 and 0.55–1, and the threshold was 0.55. In Figure 1B, the threshold for Cu^2+^ ligand was 0.52. Therefore, eight metal ion ligands were integrated, the disorder value was divided into two categories, the threshold value was set as 0.5, and the value greater than 0.5 tends to disorder. X represents the disorder value, and the classification threshold of the disorder value was represented by the function f(x).

$$f\left(x\right)=\{\begin{array}{c} I,x\in \left[0,0.5\right]\\ \mathrm{ΙΙ,x}\in (0.5,1] \end{array}$$

#### 2.2.2 Basic feature parameters

**FIGURE 2:**
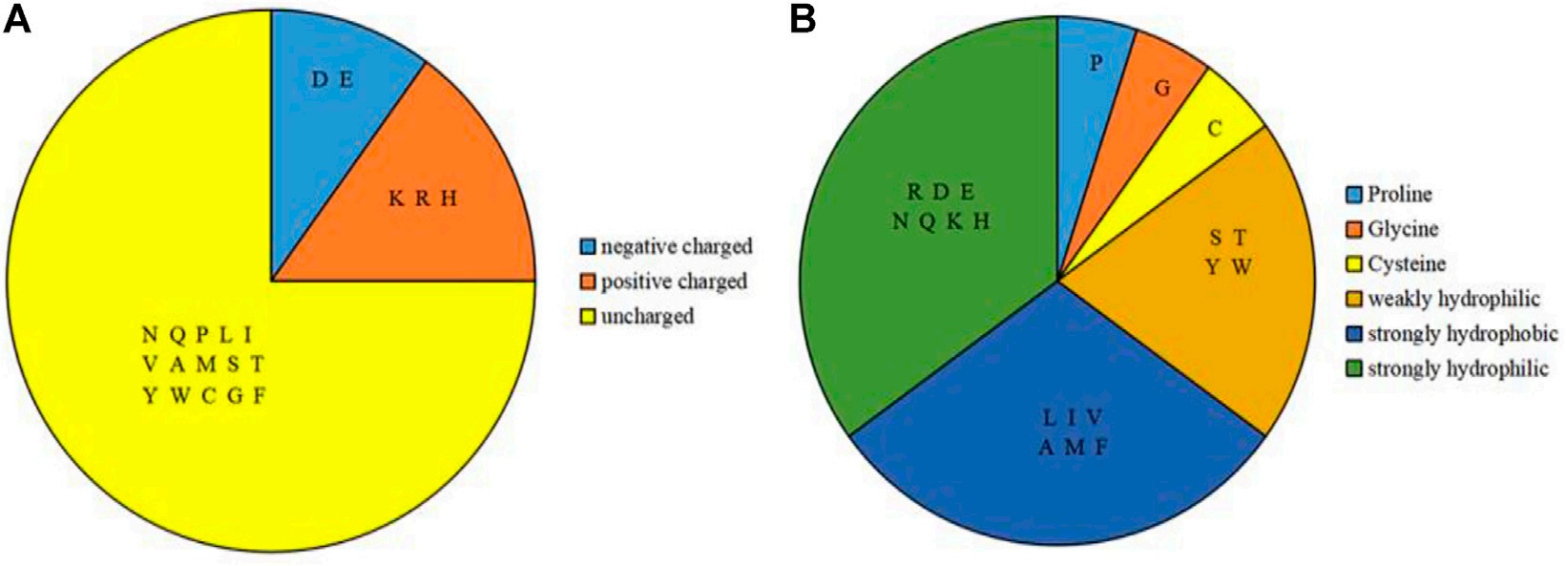
Classification of charge features and hydrophilic-hydrophobic features of amino acids. Note: **(A)** is 3 categories of the charge features; **(B)** is 6 categories of the hydrophilic-hydrophobic features.

Based on the sequence of amino acids, we selected amino acids, physicochemical features and predicted structural information as feature parameters. Among them, the physicochemical features of amino acids included the charge and hydrophilic-hydrophobic of amino acids. According to the charge properties of amino acids, the 20 amino acids were divided into three categories (Taylor, 1986), as shown in Figure 2A; according to the hydrophilic-hydrophobic properties of amino acids, the 20 amino acids were divided into six categories (Pánek et al., 2005), as shown in Figure 2B.

The predicted structural information includes: secondary structure information, relative solvent accessibility and dihedral angle (phi angle and psi angle), all of which were obtained by the ANGLOR software for protein sequences (Wu and Zhang, 2008). The secondary structure information included three types: α-helix, β-sheet and coil. According to statistical analysis, the solvent accessibility was divided into four intervals (Cao et al., 2017), and its threshold was represented by r(x):

$$r\left(x\right)=\{\begin{array}{c} \mathrm{I,x}\in (0,0.2]\\ \mathrm{II,x}\in (0.2,0.45]\\ \mathrm{III,x}\in (0.45,0.6]\\ \mathrm{IV,x}\in (0.6,0.85] \end{array}$$

The dihedral angle information was reclassified in line with statistics (Liu et al., 2020), the threshold value of the phi angle was represented by the function g(x), and the threshold value of the psi angle was represented by the function h(x):

$$g\left(x\right)=\{\begin{array}{c} I,x\in \left[-180\mathrm{{}^{\circ},-75{}^{\circ}}\right]\\ \mathrm{ΙΙ,x}\in (-75\mathrm{{}^{\circ},180{}^{\circ}]} \end{array}\;h\left(x\right)=\{\begin{array}{c} I,x\in \left[-180\mathrm{{}^{\circ},15{}^{\circ}}\right]\\ II,x\in (15\mathrm{{}^{\circ},135{}^{\circ}]}\\ III,x\in (135\mathrm{{}^{\circ},180{}^{\circ}]} \end{array}$$

### 2.3 Extraction of feature parameters

#### 2.3.1 New extraction method - propensity factors

**FIGURE 3:**
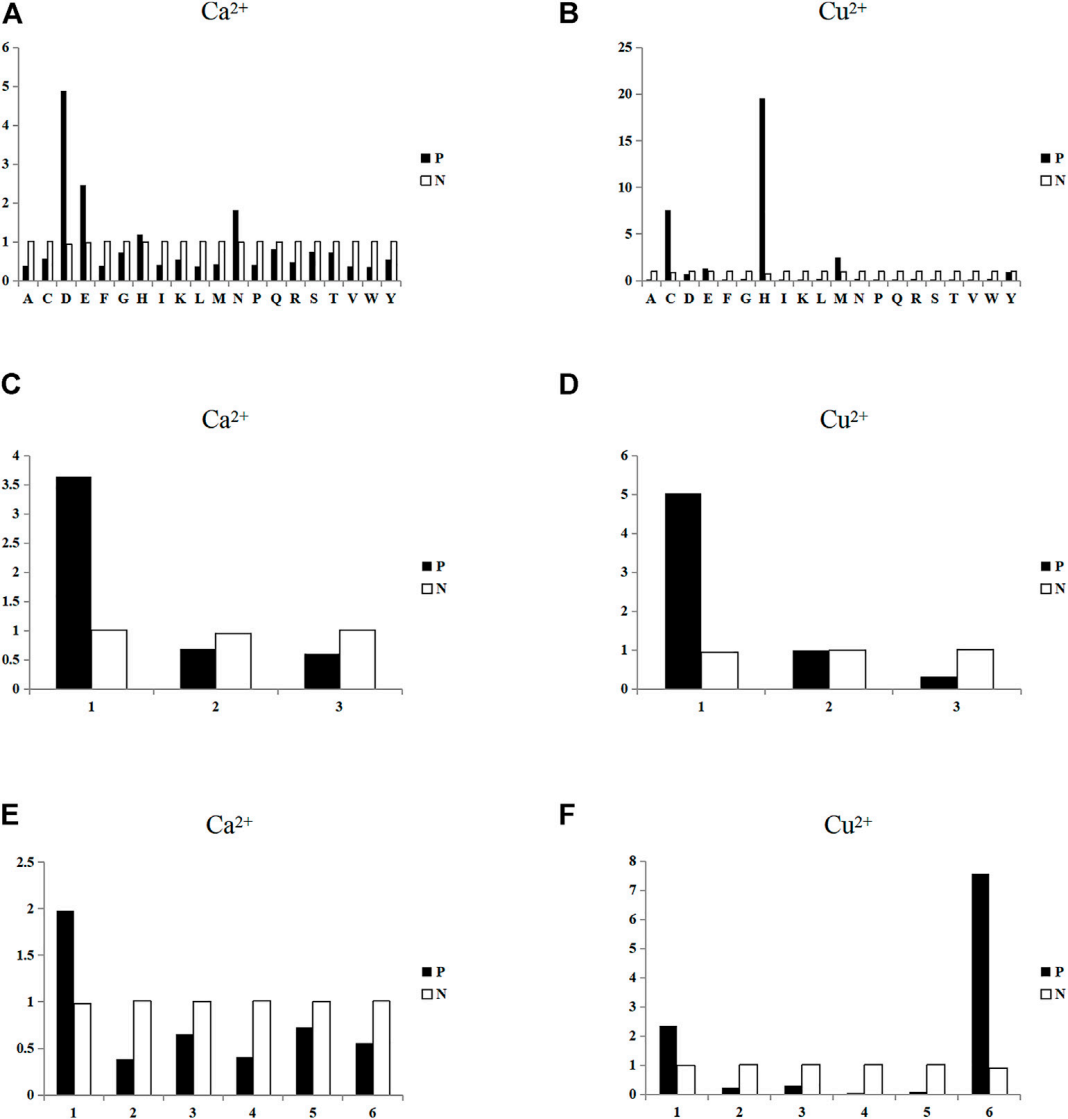
Statistical analysis of the propensity factors of binding residues and non-binding residues. Note: In Figure 3, the ordinate represents the value of propensity factors, and P and N represent binding residues and non-binding residues, respectively. Figures **(A)** and **(B)** are the statistical analysis of propensity factors of amino acids of Ca^2+^ and Cu^2+^ ligands, respectively; The abscissa represents 20 amino acids. Figures **(C)** and **(D)** are the statistical analysis of propensity factors of charge features of Ca^2+^ and Cu^2+^ ligands, respectively; and the abscissa represents the three charge classifications. Figures **(E)** and **(F)** are the statistical analysis of the propensity factors of hydrophilic-hydrophobic features of Ca^2+^ and Cu^2+^ ligands, respectively; and the abscissa represents the six hydrophilic-hydrophobic classifications.

The previous methods of extracting feature parameters were based on sequence fragments, and the effect of binding residues and their surrounding residues on the protein-ligand binding process has been sufficiently considered. However, in the process of ligand protein binding, the specific binding residues can directly interact with the ligands. The preference for amino acids and physicochemical properties of these specific binding residues has more outstanding impact on the binding process. The propensity factors first appeared in the 1970s and was proposed by two scholars, Chou and Fasman (Chou and Fasman, 1974). It has been applied to the prediction of protein secondary structure with good prediction results. The formula of the propensity factors was expressed as follow:

$$F_{ij}=\frac{p_{ij}}{p_{j}}$$ (1)

where, $p_{ij}=\frac{n_{ij}}{N_{i}}$ , $p_{j}=\frac{N_{j}}{N_{t}}$ , $N_{i}=\sum_{i=1}^{20}n_{ij}$ , $N_{t}=\sum_{j=1}^{2}N_{j}$ , *i* (*i* = 1, 2,. . ., 20) represents 20 amino acids; *j* (*j* = 1, 2) represents binding residues and non-binding residues; *n*_*ij*_ represents the number of amino acid *i* in binding residues or non-binding residues; *N*_*j*_ represents the number of binding residues or non-binding residues. Taking Ca^2+^ and Cu^2+^ as examples, the propensity factor of amino acid, charge (*i* = 1, 2, 3) and hydrophilic-hydrophobic (*i* = 1, 2,. . ., 6) were statistically analyzed, as shown in Figure 3.

In Figure 3A, for Ca^2+^ ligand, the propensity factor values of four amino acids D, E, H and N in binding residues were significantly higher than that in non-binding residues. It showed that the amino acids D, E, H, and N were more likely to be used in binding residues for Ca^2+^ ligand. Similarly, it can be seen from Figure 3B that the amino acids C, E, H, and M were more likely be used in binding residues for Cu^2+^ ligand. It can be found in Figures 3C,D that the binding residues of both Ca^2+^ and Cu^2+^ ligands tended to be positively charged. As can be seen in Figures 3E,F, the binding residues of Ca^2+^ ligands were more likely to strong hydrophilicity, and the binding residues of Cu^2+^ ligands were more likely to strong hydrophilicity and amino acid C. It can be seen from the comprehensive statistical analysis that the amino acid, charge and hydrophilic-hydrophobic had obvious preferences in binding residues and non-binding residues. Therefore, this paper used the propensity factor that can reflect the preference of binding residues as new extraction method, the above three feature parameters was extracted and used them as the predicted feature parameters. Finally, we obtain 6-dimensional propensity factor.

#### 2.3.2 Extraction method of conservative information and information entropy

The position weight matrix were widely used in the prediction of protein structure and function to extract the site conservative features, and good prediction results were obtained. Here, the position weight matrix was also used to extract the site conservative features, and the matrix elements of position weight matrix were expressed as follows (Kel et al., 2003; Gao and Hu, 2014):

$$m_{i,j}=\ln\left(\frac{p_{\mathrm{i,j}}}{p_{0\mathrm{,j}}}\right)$$ (2)

Where, $p_{\mathrm{i,j}}=\frac{\left(n_{i,j}+\frac{\sqrt{N_{i}}}{q}\right)}{\left(N_{i}+\sqrt{N_{i}}\right)}$ , $N_{i}=\sum_{j=1}^{21}n_{i,j}$ , *P*_*0,j*_ represents the background probability, and *n*_*i,j*_ represents the frequency of the *j*th amino acid at the *i*th site, *j* represents 20 kinds of amino acids and vacancies, *q* represents the number of categories, here it is 21. Two standard scoring matrices can be obtained from the positive and negative training sets, and 2L-dimensional (L is the window length) feature vector can be obtained for each segment. Similarly, the predicted secondary structure (*q* = 4), relative solvent accessibility (*q* = 5), Phi angle (*q* = 3), psi angle (*q* = 4) and disorder value (*q* = 3) were also extracted by the same method. Finally, we obtained 6*2L-dimensional the site conservative information.

According to previous studies (Liu et al., 2020; Wang et al., 2021), information entropy was used to extract charge and hydrophilic-hydrophobic and better prediction performance was obtained. Here, we also use the extraction method of information entropy. The 1-dimensional information entropy was obtained from the hydropathic-hydrophobic and charge information of amino acids, respectively. Finally, we got 2-dimensional information entropy.

The information entropy formula was expressed as (Strait and Dewey, 1996):

$$H\left(x\right)=-\sum_{j=1}^{q}p_{j}\log_{2}p_{j}$$ (3)

Where, $p_{j}=\frac{n_{j}}{N}$ , *n*_*j*_ represents the frequency of occurrence of the *j*th classification in a segment, and *N* is the segment length. For the value of *q*, if it represents the charge classification, *q* = 4; if it represents the hydrophilic-hydrophobic classification, *q* = 7.

According to previous studies (Jiang et al., 2016; Cao et al., 2017; Wang et al., 2019; Liu et al., 2020; Wang et al., 2021), it was found that good prediction results were obtained by using component information, which indicated that the component information was particularly important for predicting the binding sites of protein-metal ion ligands. Therefore, we also adopted the extraction method of component information. In the study, we extracted 21, 4, 5, 3, 4, and 3-dimensional component information for amino acids, secondary structure, relative solvent accessibility, phi angle, psi angle and disorder value, respectively. Finally, we obtained a total of 40-dimensional component information.

### 2.4 Deep neural network algorithm

Deep Neural Network (DNN) is one of the common deep learning algorithms, which aims to improve the discriminative ability of the model by providing a higher level of abstraction. Its neural network layer can be divided into input layer, hidden layer and output layer. The addition of hidden layer enhances the expression ability of the model; the extension of activation functions, such as Tanx function, Softmax function, and Relu function, etc, makes that the DNN algorithm have a wider application field. Therefore, DNN algorithm is selected as the prediction tool in this paper.

This paper used the following modules of the deep learning algorithm: the DNN backpropagation algorithm was used to train samples; the sklearn-preprocessing module was used to normalize the data; the Adam module was used as optimizer; the Relu function was used as the activation function of hidden layer; using the EarlyStopping module can effectively avoid the problem of overfitting caused by continuous training; the cross entropy loss function was used to speed up the operation. These algorithm modules were implemented under the keras framework of Python deep learning, and used TensorFlow as the back-end engine to build the DNN algorithm.

### 2.5 The validation methods and evaluation metrics

In this study, the 5-fold cross-validation was generally used to predict metal ion ligand binding residues (Hu et al., 2016b; Hu et al., 2016b; Jiang et al., 2016; Cao et al., 2017; Hu et al., 2020). For the evaluation of the prediction results, we used the methods commonly used in the prediction of protein-metal ion ligand binding residues: sensitivity (*S*_*n*_), specificity (*S*_*p*_), accuracy (*Acc*), and Matthew’s correlation coefficient (*MCC*) (Jiao and Du, 2016; Chen et al., 2019). The expressions are:

$$S_{n}=\frac{TP}{TP+FN}\times 100\%$$ (4)

$$S_{p}=\frac{TN}{TN+FP}\times 100\%$$ (5)

$$A\mathrm{cc}=\frac{TP+TN}{TP+TN+FP+FN}\times 100\%$$ (6)

$$MCC=\frac{\left(TP\times TN\right)-\left(FP\times FN\right)}{\sqrt{\left(TP+FP\right)\left(TP+FN\right)\left(TN+FP\right)\left(TN+FN\right)}}$$ (7)

In the formula, the number of metal ion ligand binding residues correctly predicted is *TP*, otherwise it is *FN*; the number of metal ion ligand non-binding residues correctly predicted is *TN*, otherwise it is *FP*.

## 3 Results and discussion

### 3.1 Prediction results of basic feature parameters

The component information (37 dimensions) and site conservative information (5*2L dimensions) of amino acids, secondary structure, relative solvent accessibility and dihedral angle, and information entropy (2 dimensions) of charge and hydropathic-hydrophobic were fused as feature parameters, the DNN algorithm was used to predict, and the 5-fold cross-validation results were shown in Table 2 (DNN^a^). Overall, the predicted results were not ideal. The S_n_ value of the eight metal ion ligands was only over 11.53%, the S_p_ and Acc values were only better than 96.38%, and the MCC value was only better than 0.1354.

**TABLE 2:** Comparison of 5-fold cross-validation results.

Ligand	Algorithm	Hidden layers	Hidden neurons	Batch size	Sn(%)	Sp(%)	Acc(%)	MCC
Zn2+	DNNa	2	64	64	26.65	99.34	98.21	0.3147
	DNNb	2	64	64	31.49	99.51	98.45	0.3923
	DNNc	2	16	16	33.33	99.73	98.69	0.4630
	IonSeq	—	—	—	43.56	99.21	99.75	0.5043
Cu2+	DNNa	2	64	64	38.97	98.62	97.78	0.3237
	DNNb	2	64	64	42.06	99.07	98.27	0.3982
	DNNc	4	64	16	49.90	99.38	98.68	0.5070
	IonSeq	—	—	—	50.65	99.01	99.69	0.5868
Fe2+	DNNa	2	64	64	29.32	98.74	97.85	0.2504
	DNNb	2	64	64	33.25	99.15	98.30	0.3264
	DNNc	2	16	16	35.84	99.27	98.45	0.3659
	IonSeq	—	—	—	54.08	99.51	98.84	0.5772
Fe3+	DNNa	2	64	64	27.27	99.47	98.32	0.3254
	DNNb	2	64	64	29.29	99.49	98.39	0.3452
	DNNc	2	16	16	32.08	99.51	98.49	0.3953
	IonSeq	—	—	—	52.27	99.81	99.21	0.6370
Co2+	DNNa	2	64	64	11.53	99.18	97.81	0.1354
	DNNb	2	64	64	16.00	99.36	98.06	0.2051
	DNNc	4	16	16	17.83	99.37	98.10	0.2254
	IonSeq	—	—	—	—	—	—	—
Mn2+	DNNa	2	64	64	15.74	99.71	98.60	0.2462
	DNNb	2	64	64	17.62	99.70	98.61	0.277
	DNNc	3	16	32	18.17	99.74	98.65	0.2933
	IonSeq	—	—	—	31.07	99.82	99.01	0.4553
Ca2+	DNNa	2	64	64	20.42	98.52	97.20	0.1831
	DNNb	2	64	64	26.46	98.68	97.42	0.2315
	DNNc	2	32	32	28.14	98.72	97.62	0.2664
	IonSeq	—	—	—	22.72	99.04	98.18	0.2111
Mg2+	DNNa	2	64	64	22.85	96.38	96.67	0.1852
	DNNb	2	64	64	32.85	98.33	97.61	0.2291
	DNNc	4	64	32	34.82	98.52	97.83	0.2565
	IonSeq	—	—	—	5.57	99.98	99.49	0.1825

Note: DNN^a^, is the prediction result of without optimization of hyper-parameters and without adding disorder value and propensity factor; DNN^b^, is the prediction result of without optimization of hyper-parameters and adding disorder value and propensity factor; DNN^c^, is the prediction result of optimization of hyper-parameters and adding disorder value and propensity factor; IonSeq is data obtained from Reference (Hu et al., 2016b).

### 3.2 Prediction results of adding disordered value and propensity factors

**FIGURE 4:**
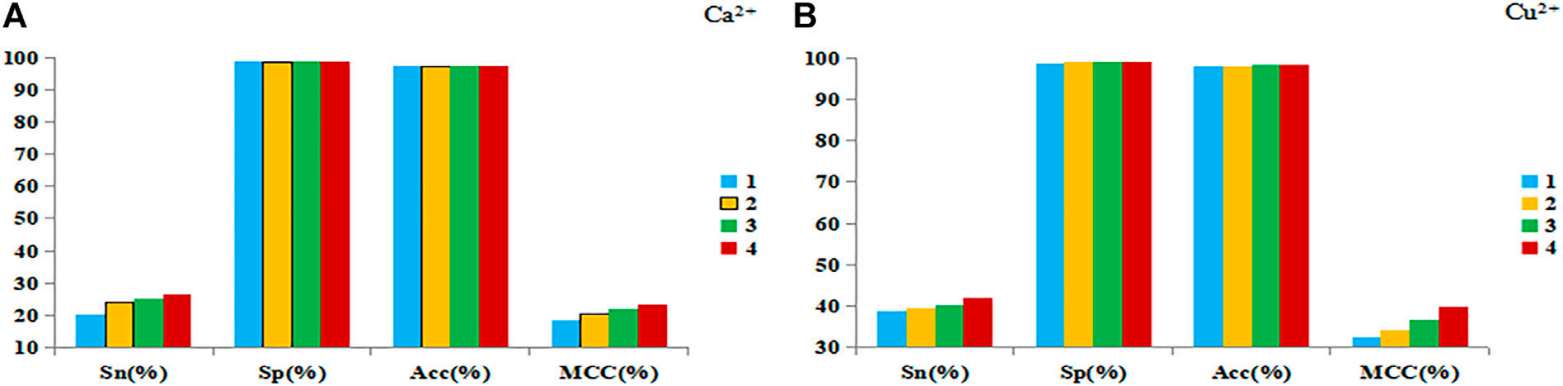
The results of 5-fold cross-validation of Ca^2+^ **(A)** and Cu^2+^ **(B)** ligands. Note: The abscissa is the four evaluation indexes, and the ordinate is the value of the evaluation index. The ordinate is the value of the evaluation index. The blue bar represents the prediction results of the basic feature parameters, the yellow bar represents the prediction results of 1+propensity factor, the green bar represents the prediction results of 1+disorder value, and the red bar represents the prediction results of 2+ disorder value.

In order to further improve the prediction performance, disorder value and propensity factor were introduced, and the DNN algorithm was used to predict the metal ion ligand binding residues. The results of 5-fold cross-validation of Ca^2+^ and Cu^2+^ ligands as examples were shown in Figure 4.

It can be found from Figures 4A,B that when the disorder value and propensity factor were added separately, the S_n_ and MCC values were significantly improved, and the S_p_ and Acc values were almost unchanged. When the disorder values and propensity factor were used at the same time, the prediction results were the best. Therefore, we believed that both the disorder value and the propensity factor had a more positive effect on the prediction of metal ion ligand binding residues.

The prediction results of feature parameters four are listed in Table 2 (DNN^b^). It can be seen from Table 2 that the S_n_ value of the eight metal ion ligands reached 16%, the Sp and Acc values reached 97.42%, and the MCC value reached 0.2051. Compared with the prediction results of the basic parameters, it can be found that all the four evaluation indexes of the eight kinds of ions have been improved, in which the S_n_ and MCC values increased significantly. For example, the Sn value of Mg^2+^, Ca^2+^, Co^2+^ and Zn^2+^ ligands increased by 10%, 6.04%, 4.47% and 4.84%, respectively; the MCC value increased by 0.0439, 0.0484, 0.0697 and 0.0776, respectively. It can be seen that the adding disordered value and propensity factor can effectively improve the prediction performance.

### 3.3 Optimization of hyper-parameters

The hyper-parameters of deep learning algorithms include: learning rate, activation function, and number of epochs, etc. The hyper-parameters had great influence on the training speed and performance of the predictor. Therefore, we optimize the hyper-parameters to improve the prediction performance. Considering the influence on model accuracy, computing resources, computing time and previous studies (Koutsoukas et al., 2017), we selected three hyper-parameters to optimize, which included the number of hidden layers, the number of hidden layer nodes (the number of hidden neurons) and the batch size. The value range of the optimized hyper-parameters was given in Table 3.

**TABLE 3:** Value range of hyper-parameters.

Hyper-parameters	Value range
Hidden layers	1,2,3,4,5,6,7,8
Hidden layer nodes	2,4,8,16,32,64,128
Batch size	2,4,8,16,32,64,128

**FIGURE 5:**
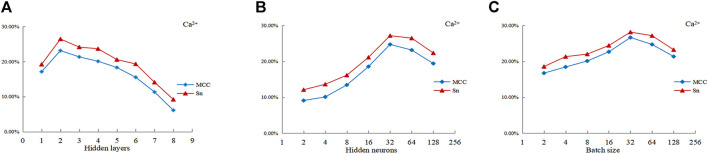
Curve of MCC value and S_n_ value of Ca^2+^ ligands with hyper-parameters. Note: The abscissas of **(A-C)** represent three hyperparameters, respectively. The ordinate is the value of MCC and S_n_; MCC and S_n_ are the evaluation index.

Taken Ca^2+^ as examples, Figure 5A is a line chart showing the MCC value and Sn value of Ca^2+^ ligands with the number of hidden layers. It can be seen from Figure 5A that the number of hidden layers had great influence on the performance of the predictor. When the number of hidden layers was 2, both the MCC and Sn values reached their peaks. We took the optimal layer value for Ca^2+^ ligand as 2. From Figure 5B, it can be known that the optimal hidden layer node value of Ca^2+^ ligands was 32. From Figure 5C, it could be seen that the optimal batch size of Ca^2+^ ligands was 32.

The prediction results after optimization of hyper-parameters were shown in Table 2 (DNN^c^). It can be seen from the results that the S_n_ value of eight metal ion ligands reached 17.83%, the S_p_ and Acc values reached 97.62%, and the MCC value reached 0.2254. Compared with DNN^b^, it was found that the optimization of hyper-parameters could effectively improve the prediction performance of the DNN algorithm, and the four evaluation indexes had a certain improvement. The S_n_ and MCC values were significantly improved, in which the S_n_ value of Cu^2+^ and Fe^3+^ ligands increased by 7.84 and 2.79%, respectively. The MCC value of Fe^3+^, Cu^2+^ and Zn^2+^ ligands increased by 0.0501, 0.1088 and 0.0707, respectively.

### 3.4 Comparison of predicted results

In order to verify the reliability and practicability of the prediction model, the results were compared with the previous IonSeq method. For the convenience of comparison, the results of the IonSeq method were also listed in Table 2. Through analysis and comparison, it was found that the evaluation index of the prediction result of the DNN algorithm has the same characteristics as the IonSeq method. Both the methods have small S_n_ value and large S_P_ value. The reason for this result was that the number of negative samples was much greater than that of positive samples in the dataset. The results of DNN algorithm for alkaline Earth metals (Mg^2+^ and Ca^2+^) were better than IonSeq method, in which the S_n_ and MCC values of Mg^2+^ ligand increased by 29.25% and 0.074, respectively. The S_n_ and MCC values of Ca^2+^ ligand increased by 5.42% and 0.0553, respectively. The prediction results of Cu^2+^ ligand were closest to the IonSeq method, and the S_p_ value was slightly higher than the IonSeq method. Co^2+^ ligand can’t be compared with the IonSeq method, but the prediction performance was greatly improved by comparing the prediction results. The prediction results of the other four metal ion ligands using the DNN algorithm were slightly poor. Although not all of our results were better than the IonSeq method, the DNN algorithm had a certain positive effect on the prediction of metal ion ligand residues.

## 4 Conclusion

In this paper, based on the information of protein sequence and sequence-derived structure, the DNN algorithm was used to predict eight types of metal ion ligands binding residues. The introduction of new feature parameters and extraction methods perfected the basic feature parameter information, which helped to identify metal ion ligand binding sites and improved the prediction performance. The hyper-parameter optimization of the model effectively improved the prediction performance of the DNN model. In comparison with IonSeq, the obtained prediction model based on sequence information, sequence-derived structure information and DNN algorithm was not very perfect. However, in view of the universality and practicability of the prediction model, DNN model can be used as a supplementary model to predict metal ion ligand residues.

## Data Availability

The original contributions presented in the study are included in the article/supplementary material, further inquiries can be directed to the corresponding authors.
